# Prospective pharmacological methodology for establishing and evaluating anti-cancer drug resistant cell lines

**DOI:** 10.1186/s12885-021-08784-7

**Published:** 2021-09-25

**Authors:** Hoon Yu, Dong-Jin Kim, Hye-Young Choi, So Myoung Kim, Md. Intazur Rahaman, Young-Hoon Kim, So Won Kim

**Affiliations:** 1 Division of Nephrology, Department of Internal Medicine, GangNeung Asan Hospital, University of Ulsan College of Medicine, Gangneung, Republic of Korea; 2 Drug Evaluation Department, National Institute of Food and Drug Safety Evaluation, Ministry of Food and Drug Safety, Osong, Cheongju, Republic of Korea; 3 Department of Pharmacology, Catholic Kwandong University College of Medicine, Gangneung, Republic of Korea; 4 Department of Pharmacology, Asan Medical Center, University of Ulsan College of Medicine, 88, Olympic-ro 43-gil, Songpa-gu, Seoul, Republic of Korea; 5 Bio-Medical Institute of Technology, University of Ulsan, Seoul, Republic of Korea

**Keywords:** Renal cell carcinoma, Sunitinib, Drug resistance, Cross-resistance, IC_50_, GR_100_

## Abstract

**Background:**

Cell lines are often used to assess the resistance of anticancer drugs when in vivo analysis is not possible. However, the process for establishing anti-cancer drug resistance in cell cultures in vitro and the subsequent method of then evaluating resistance are not clearly established. Traditionally, the IC_50_ is the most commonly used indicator of resistance evaluation but it cannot represent the effectiveness of anti-cancer drugs in a clinical setting and lacks reliability because it is heavily affected by the cell doubling time. Hence, new indicators that can evaluate anti-cancer drug resistance are needed.

**Methods:**

A novel resistance evaluation methodology was validated in this present study by establishing sunitinib resistance in renal cell carcinoma cells and assessing the cross-resistance of five different anti-cancer drugs.

**Results:**

It was confirmed in this present study that the IC_50_ does not reflect the cell proliferation rates in a way that represents anti-cancer drug resistance. An alternative indicator that can also be clinically meaningful when using in vitro cell line systems is GI_100_. Additionally, the GR_100_ allows different cell populations to be calibrated on the same basis when multiple experimental results are compared.

**Conclusion:**

Since the GR_100_ has properties that indicate the efficiency of anti-cancer drugs, both the efficacy and GR_100_ of a particular anti-cancer drug can be used to effectively assess the resistance.

**Supplementary Information:**

The online version contains supplementary material available at 10.1186/s12885-021-08784-7.

## Background

Human in vivo research systems would always yield the most accurate results when testing a clinical therapy, but are problematic to establish due to factors such as accessibility, risks, and ethical considerations, among others. Safer and simpler research systems that can somewhat reflect the clinical environment are therefore almost always used. It is vital in this circumstance that the research design should be as similar as possible to the clinical environment and that any in vitro data thereby obtained should be at a level that can be given serious consideration in a clinical setting. The cancer field is no exception and immortalized cell lines originally derived from human tissues are commonly used to conduct cancer research. Many studies have also established cells that are resistant to anti-cancer drugs using the IC_50_ as an indicator of resistance assessment without criticism [1–3]. It must be noted however that the IC_50_ measure has many weaknesses and is not actually a suitable indicator of the resistance of anti-cancer drugs. It thus remained necessary to establish a methodology for evaluating the resistance of anti-cancer drugs using in vitro cell systems and any such approach would need to be designed so that the findings could be applied to a clinical context.

**Fig. 1:**
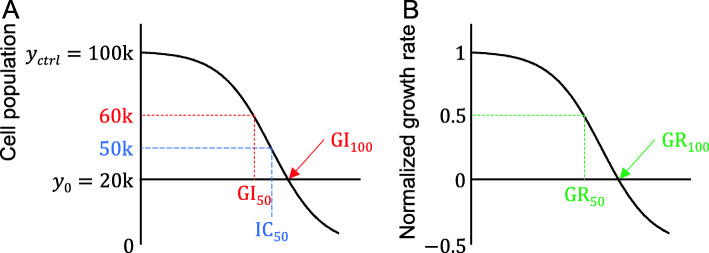
Candidate indicators of anti-cancer drug resistance in cell lines. **A** The graph indicates anti-cancer drug treatment of cells, and measures the number of living cells, over a certain period of time. The y-axis refers to the number of cells and the x-axis refers to the concentration of anti-cancer drugs. y_0_ is the number of cells just before exposure to the anti-cancer drug, and y_ctrl_ is the number of cells after a certain period of time in the control group of cells that are not treated. IC_50_ refers to the drug concentration when the number of cells is 50% of that of the y_ctrl_, regardless of the value of y_0_. GI refers to the inhibition of cell proliferation so that the (*y*_*ctrl*_ − *y*_0_) value becomes a 100% cell proliferation rate. GI_50_ is the concentration of the drug at which cell proliferation is reduced by 50%, i.e. the concentration at which the number of cells is $\left({y}_0+\frac{y_{ctrl}-{y}_0}{2}\right)$, and GI_100_ is the concentration at *y*_0_. **B** In graph A, replacing the cell population with a normalized growth rate takes into account the cell doubling time, resulting in a y-axis value of between − 1 and 1. At this point, the drug concentration corresponding to the y-axis value of 0.5 is the GR_50_, and the concentration corresponding to 0 is the GR_100_. This figure is an adaptation of a figure published previously by Brooks et al. [8]

The criteria for judging the effectiveness of anti-cancer drugs is a principal consideration in the design of any new methodology to measure resistance and can be ultimately summarized as whether a reduction in tumor size has been achieved [4–6]. Formal guidelines classify anticancer effects by considering the margin of error and specifying the degree of tumor size change [7]. In cell experiments, the tumor size change corresponds to a change in the number of cells and is usually shown on the y-axis of the graph as a dependent variable against the drug concentration. The IC_50_ has no clinical significance in this type of experiment because it indicates the concentration of a drug that has caused a 50% reduction in cell number compared to a control group that has not been treated (Fig. 1A). To have clinical meaning in terms of the anti-cancer effectiveness of a given drug, the number of cells should be equivalent or less than that prior to treatment (y_0_ and less in Fig. 1A). This can be determined by evaluating the growth rate from the seeding cells, and utilizing the GI instead of the IC (Fig. 1A) [9]. Additionally, the IC can vary if cell doubling times are different. To address this, the GR system is a method of representing GI by calibrating the doubling time problem associated with the IC (Fig. 1B) [8,10].

A new and effective anti-cancer drug resistance evaluation system is described and validated in this present study. In the analyses, the IC, GI, and GR indicators were compared by establishing cells that are resistant to sunitinib, one of the first choice drugs used to treat renal cell carcinoma (RCC). Only resistance assessment indicators before and after the establishment of resistant cells has been compared in prior studies whereas this present investigation observed changes in resistance assessment indicators over a long period during which resistance was established to verify the utility of resistance assessment indicators. After the establishment of sunitinib-resistant cells, their cross-resistance to five anti-cancer drugs was evaluated and these indicators were compared again.

## Methods

### Cell culture conditions

For this study, cells with a clear cytology, which includes the majority of RCCs, and short doubling times were preferred [8]. The SNU-228 and SNU-267 lines were thus chosen (00228 and 00267, Korean Cell Line Bank) and maintained in RPMI 1640 medium (LM011–03, Welgene) supplemented with 10% fetal bovine serum (FBS, 16000044; Gibco). The cells were incubated at 37 °C in a 5% CO_2_ humidified incubator. To establish sunitinib resistance, the methods described by Martina et al. were referred to [3]. Briefly, the cells were exposed to sunitinib on a six-week cycle. Reflecting the clinical administration of sunitinib, the drugs were administered for 4 weeks and then withdrawn for 2 weeks. After the cells were exposed to the test drug for 4 weeks, the concentration of the drug was halved every 48 h with consideration of the 40 to 60 h half-life in the body, to simulate the clinical two-week withdrawal period [11]. The drug concentrations used are described in Supplementary Table 1. The cells were continuously exposed to culture medium containing the drug, even during subculture, except when viability measurements were conducted in 96-well plates.

### Chemicals

The drugs used in this study were as follows: sunitinib (SYN-1086-M001, Adipogen Life Sciences); axitinib (SYN-1014-M010, Adipogen); cabozantinib (S1119, Selleckchem); pazopanib (CDS023580-25MG, Sigma Aldrich, St. Louis, MO); sorafenib (AG-CR1–0025-M005, Adipogen); everolimus (ab142151, Abcam); and temsirolimus (PZ0020-5MG, Sigma).

### Cell viability measurement

Live cells were quantitatively measured using an EZ-CYTOX cell viability, proliferation, & cytotoxicity assay kit (EZ-3000, DoGenBio) in accordance with the manufacturer’s instructions. Briefly, SNU-228 and SNU-267 cells were seeded in a 96-well plate at a density of 1000 and 2000 cells, and in 100 μl media per well, respectively. At 24 h after cell seeding, the tested drugs were added over a concentration gradient. EZ-CYTOX solution (10 μl) was added to the cells at 72 h after the drug exposure. After 1 h of incubation at room temperature, the plates were gently shaken to mix the contents for 30 s, and cell viability was estimated by the absorbance readings at a 450 nm wavelength, measured using a microplate reader.

### Usage indicators for evaluating the effects of the drugs on cell survival

The IC_50_, GI_50_, GI_100_, GR_50_, and GR_100_ were calculated in the experiments, all among the known indicators of drug efficacy (Fig. 1) and that are defined as follows [8]. The IC_50_ denotes the concentration of a drug that causes a reduction in the cell population to half the number at zero time (i.e. the point immediately before the drug is added). The GI_50_ is the drug concentration that produces a reduced cell growth from seeding to 50% of the maximum, and the GI_100_ reflects the drug concentration at which the number of seeding states is maintained. When the y-axis is normalized by taking into account the cell doubling time, the drug concentration at the normalized counts of 0.5 and 0 is defined as the GR_50_ and GR_100_, respectively. In this present study, the GR_50_ and GR_100_ values were calculated using the previously described fixed-interval formula as follows [10]:

$$ GR(c)={2}^{\frac{\log_2\left({\mathcal{x}}_{(c)}/{\mathcal{x}}_0\right)}{\log_2\left({\mathcal{x}}_{ctrl}/{\mathcal{x}}_0\right)}}-1 $$

### Statistical analysis

Trends with regards to increasing or decreasing indicators related to cell growth or drug reactions were analyzed by linear regression, and 95% confidence intervals were calculated. Statistical significance was determined by a *p*-value of 0.05 or less.

## Results

### Process for establishing resistant cells

**Fig. 2:**
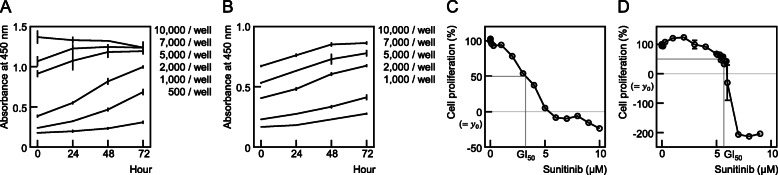
Determining the appropriate number of cells and the sunitinib concentration required to generate sunitinib-resistance in SNU-228 and SNU-267 cells. Cell proliferation was measured for 72 h after seeding the cells at 500, 1000, 2000, 5000, 7000, and 10,000 cells/well in 96-well plates; **A** SNU-228 and **B** SNU-267 cells. The y-axis is the absorbance measured at 450 nm (A and B). The population of SNU-228 cells was measured after 1000 cells/well were seeded in 96-well plates and then exposed to a concentration gradient of sunitinib for 72 h (**C**). SNU-267 cells were tested in the same way after 2000 cells/well were seeded (**D**). The GI_50_ values were then calculated. This experiment was conducted three times independently

In order to properly measure cell viability following exposure to an anti-cancer drug, the number of cells seeded onto 96-well plates needs to be accurately determined in advance. This is to ensure that cells are not affected by effects other the drug exposure when proliferating. SNU-228 cells were seeded at a density of 500–10,000/well and their viability was measured every 24 h. For the 500 and 1000 cells/well densities, the slope of the graph continues to increase until 72 h. However, in terms of the number of cells, the slope of the graph decreased from 48 h. This indicated that the proliferation had slowed from the 48 h timepoint due to cell saturation. At a 2000 cells/well density for the SNU-267 line, there were no affects of cell saturation on the increases in proliferation. Hence, the experiments using SNU-228 cells were conducted at 1000/well and using SNU-267 at 2000/well in a 96-well plate to eliminate any confounding impacts of cell saturation (Fig. 2A, B). When growing cells in a larger plate or flask, the area ratio in relation to the 96-well plate was calculated and the cells were seeded accordingly.

Once the appropriate number of cells to seed has been determined, the concentration of the drug to add to culture medium must be determined to establish resistance. The concentrations used in clinical settings are preferable in this regard, and the drug dosage and blood concentration are the available parameters. The blood concentration is not particularly helpful however because it differs from the target organ concentration. The concentration in the present experiments was thus determined based on the cell responses to the drug. However, treatments with drugs at concentrations that can reduce the number of cells targeted in a clinical setting (y_0_ or higher) is not easy to maintain for long periods of time in a laboratory. In this current study, the concentrations that reduced cell growth by 50% were used (i.e. the GI_50_). The GI_50_ value was measured at 2.97 μM for SNU-228 cells and 5.67 μM for SNU-267 cells, and it was finally decided to expose these cell types to 3 μM and 5.5 μM doses, respectively, for convenience (Fig. 2C, D).

### Process for establishing sunitinib-resistant cells

**Fig. 3:**
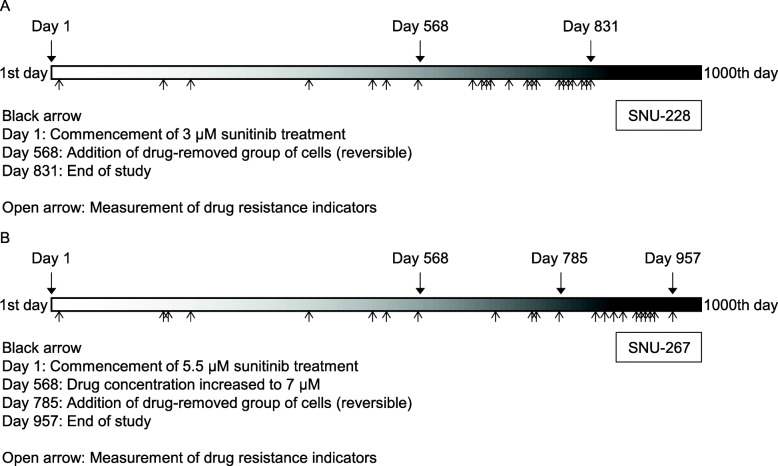
Schematic representation of the sunitinib-resistant cell line establishment process. The schema timeline is from the day on which the cells were exposed to sunitinib up to 1000 days later. SNU-228 cells (**A**) were eventually exposed to sunitinib for 831 days and SNU-267 cells (**B**) for 957 days. Closed arrows highlight exposure to the drugs, and open arrows indicate when sunitinib resistance was measured

To establish drug resistance, the SNU-228 cells were exposed to 3 μM sunitinib for a total of 831 days. Sunitinib is clinically used over a 6 week cycle (a two-week break after a four-week treatment), and the cell cultures were treated accordingly and the drug half life was considered (Supplementary Table 1). From day 568 after the initial exposure of this cell line, a group of cells that had been drug-removed was added to determine whether the onset of resistance was reversible. The SNU-267 cells were exposed to sunitinib for a total of 957 days. Sunitinib exposure was initiated at a dose of 5.5 μM, and the concentration was increased to 7 μM on the day 568. A group of drug-removed cells was added on day 785 (Fig. 3).

### Trends in the occurrence of resistance and comparison of resistance indicator candidates after exposure to sunitinib

**Fig. 4:**
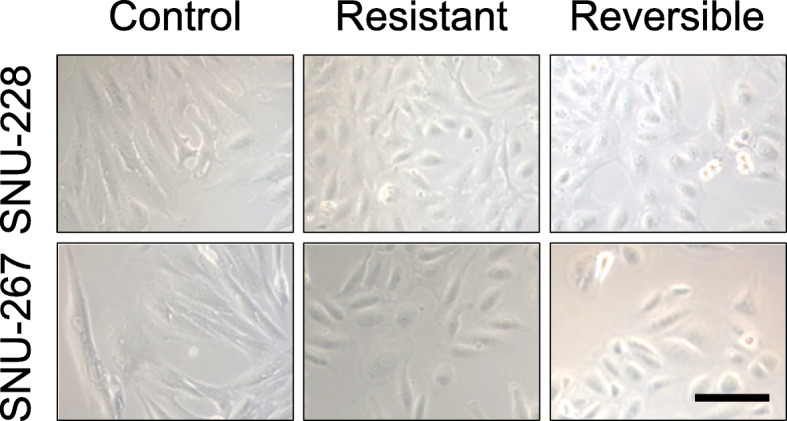
Changes in the SNU-228 and SNU-267 morphologies due to sunitinib exposure. The shapes of the cells were monitored in the control, sunitinib-resistant, and sunitinib removal cells. Scale bar, 100 μm

To confirm the establishment of resistance in the two RCC cell lines used in this study, their cellular appearance was first observed. It was possible to confirm that morphological changes had occurred in the cells after the sunitinib treatment regimen was fully completed. The control cells showed an elongated shape for both the SNU-228 and SNU-267 lines, whereas the sunitinib-treated cells were much shorter. Even in the treated cells that had been sunitinib free for 3 months since resistance was established, the control cell shape was not restored (Fig. 4).

After exposure of the two RCC cell lines to sunitinib, as shown in Fig. 3, cell viability were measured and resistance indicators were evaluated. The establishment of resistance was confirmed by linear regression indicating a straight line of resistance indicators over time. The results of the linear regression analysis for all resistance indicators are presented in Table 1 for SNU-228 cells and in Table 2 for SNU-267 cells.

**Table 1:** Simple linear regression analysis of resistance indicator candidates using sunitinib-treated SNU-228 cells

		Equation	95% confidence interval of slope	*p* value
Growth rate	Control cells	*y* = 0.04988*x* + 134.9	−0.04465 to 0.1444	0.2863
	Resistant cells	*y* = 0.2151*x* + 66.28	0.1348 to 0.2954	< 0.0001
IC_50_	Control	*y* = 0.002971*x* + 5.56	−0.004794 to 0.01074	0.3957
	Resistant cells	*y* = 0.00237*x* + 6.11	−0.003783 to 0.008524	0.3077
GI_50_	Control	*y* = 0.000812*x* + 2.484	−0.002979 to 0.004603	0.6491
	Resistant cells	*y* = 0.00326*x* + 2.838	−0.00002263 to 0.006543	0.0513
GI_100_	Control	*y* = 0.006882*x* + 4.776	−0.0003099 to 0.01407	0.0588
	Resistant cells	*y* = 0.04409*x* − 7.048	0.025 to 0.06317	0.0019
GR_50_	Control	*y* = 0.001119*x* + 2.339	−0.002605 to 0.004843	0.5251
	Resistant cells	*y* = 0.003732*x* + 2.54	0.0005129 to 0.00695	0.0269
GR_100_	Control	*y* = 0.006869*x* + 4.79	−0.0003287 to 0.01407	0.0594
	Resistant cells	*y* = 0.04408*x* − 7.043	0.025 to 0.06317	0.0019

**Table 2:** Simple linear regression analysis of resistance indicator candidates using sunitinib-treated SNU-267 cells

		Equation	95% confidence interval of slope	*p* value
Growth rate	Control	*y* = 0.04181*x* + 244.4	−0.1577 to 0.2414	0.6668
	Resistant cells	*y* = 0.4506*x* + 107.4	0.2190 to 0.6821	0.0007
IC_50_	Control	*y* = 0.0006978*x* + 5.224	−0.001398 to 0.002793	0.4847
	Resistant cells	*y* = 0.0003528*x* + 6.639	−0.002357 to 0.003063	0.7798
GI_50_	Control	*y* = 0.0004711*x* + 4.34	−0.001095 to 0.002037	0.5293
	Resistant cells	*y* = 0.001636*x* + 4.439	−0.0005583 to 0.00383	0.1303
GI_100_	Control	*y* = − 0.0001539*x* + 6.351	−0.00131 to 0.001002	0.7805
	Resistant cells	*y* = 0.007164*x* + 4.328	0.004733 to 0.009596	< 0.0001
GR_50_	Control	*y* = 0.000252*x* + 4.646	−0.0008375 to 0.001342	0.6275
	Resistant cells	*y* = 0.002473*x* + 4.427	0.0004343 to 0.004512	0.0215
GR_100_	Control	*y* = − 0.0003388*x* + 6.453	−0.001525 to 0.000847	0.5516
	Resistant cells	*y* = 0.007258*x* + 4.248	0.004843 to 0.009674	< 0.0001

**Fig. 5:**
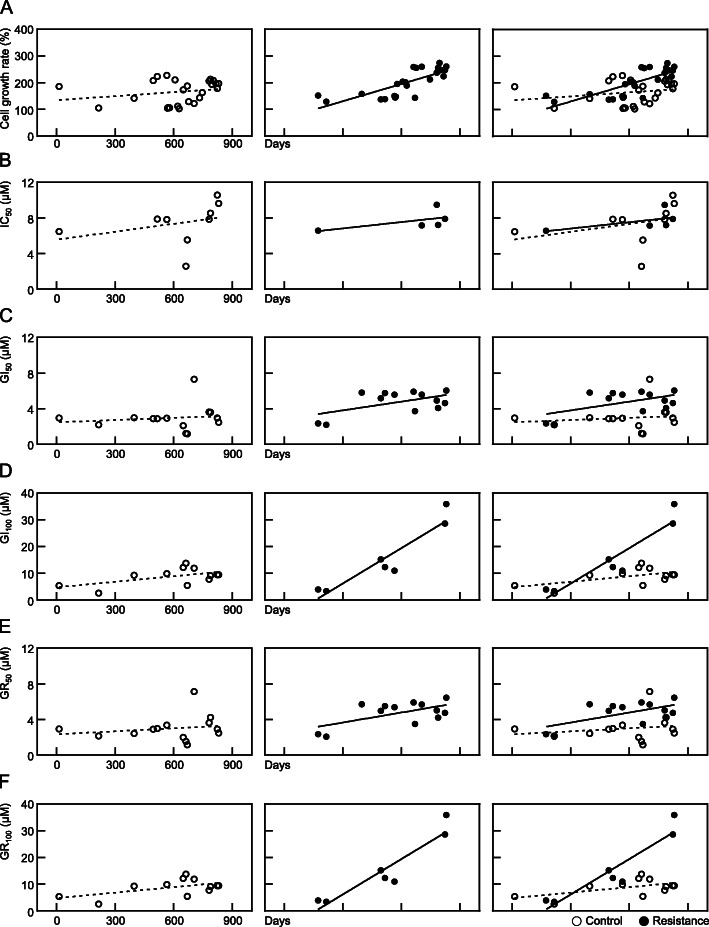
Trends for cell growth and resistance indicator candidates in SNU-228 cells in accordance with the duration of exposure to sunitinib. The cell growth rate and resistance indicator candidates in SNU-228 cells measured on the dates indicated by the open arrows in Fig. 3 are displayed graphically by date. Data from the control groups not exposed to drugs are shown in the left column, the resistant groups exposed to sunitinib are shown in the middle column, and the two groups combined are shown in the right column. The results of simple linear regression analysis are shown in a straight line and the 95% confidence interval is also shown in the left and middle columns

**Fig. 6:**
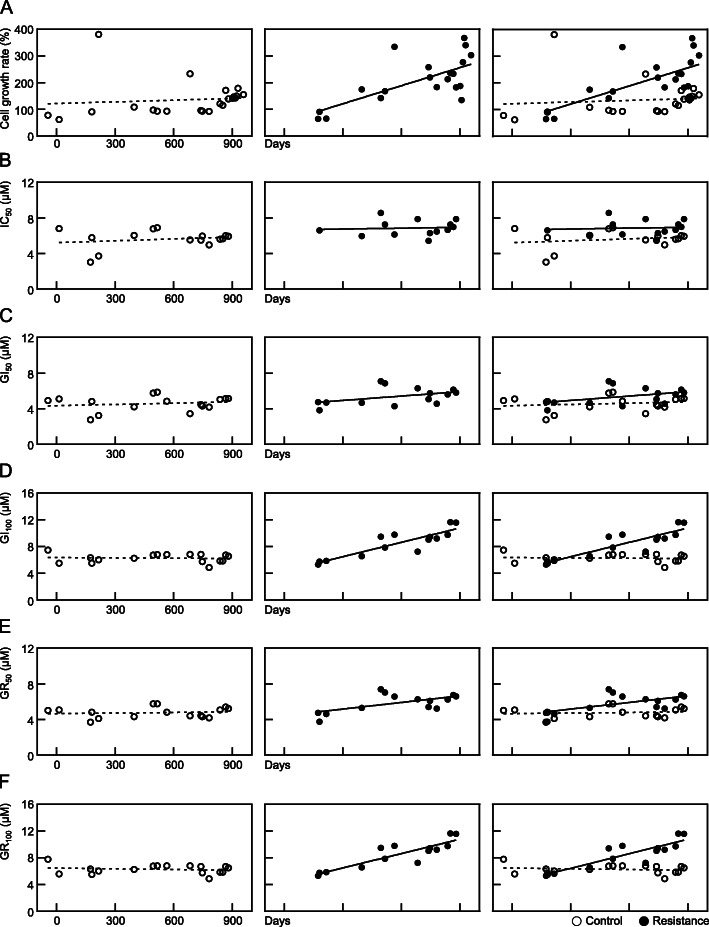
Trends for cell growth and resistance indicator candidates in SNU-267 cells in accordance with the duration of exposure to sunitinib. Identical experiments to those described in Fig. 5 were conducted in SNU-267 cells

The factor that was found to best represent resistance to anti-cancer drugs was changes in cell proliferation. The growth rate of the control SNU-228 cells was measured for 72 h and listed according to date. The growth rate trend of the control cells showed no statistically significant increase (*p* = 0.2863) whereas the sunitinib-exposed cells had a statistically significantly increased rate (*p* < 0.0001, Fig. 5A). Similar results were obtained for the SNU-267 cells. Again, the cell growth rate of the control group cells was not statistically different (*p* = 0.6668) whereas that of the sunitinib-treated cells increased (*p* = 0.0007, Fig. 6A).

The trends in terms of IC_50_ values, which are calculated based on the total number of cells and are the most utilized measure of drug activity, were next analyzed. The SNU-228 cells showed no statistically significant pattern of increase in this value in either the control or sunitinib-treated cells (*p* = 0.3957 and 0.3077, Fig. 5B). In the SNU-267 cells also, there was no pattern of increase in the IC_50_ values in either the control cells (*p* = 0.4847) or sunitinib-treated cells (*p* = 0.7798, Fig. 6B). There were some instances at particular timepoints in which the IC_50_ of the sunitinib-treated cells was higher than that of the control cells (i.e. 1.42-fold higher at 684 days after sunitinib treatment in the SNU-267 cells). However, the analyses over the entire study period indicated no specific increases compared to the control cells. Hence, the IC_50_ values did not reflect the process of establishing resistance.

The GI_50_ is the second most commonly used parameter after the IC_50_ for assessing the effectiveness of an anti-cancer drug, and is calculated on a growth rate basis, not on the highest number of cells. The GI_50_ values in sunitinib-treated SNU-228 cells seemed to differ from the control cells over time, but this was not statistically significant (*p* = 0.6491 for control and *p* = 0.0513 for sunitinib treated cells, Fig. 5C). Similarly, there was no statistical increase in the GI_50_ in either the control or sunitinib-treated SNU-267 cells (*p* = 0.5293 and 0.1303, Fig. 6C).

The GI_100_ parameter is a concept that adds clinical significance to the concentration at which cell growth is at least 100% inhibited. There was no statistical increase in the GI_100_ values of the SNU-228 control cells (*p* = 0.0588), but this measurement was increased in the sunitinib-treated cells (*p* = 0.0019, Fig. 5D). A similar pattern appeared in SNU-267 cells, i.e. there was no increase in the SNU-267 control cells (*p* = 0.7805) but a significant increase was found in the sunitinib-treated cells (*p* < 0.0001, Fig. 6D). In addition, and unlike the GI_50_, the trend in relation to the GI_100_ values showed statistical differences between the control cells and sunitinib-treated cells.

The GR_50_ results in this study, a measure which was developed previously by Hafner and colleagues that takes account of the cell doubling time as an indicator of an anti-cancer drug’s effectiveness, were confirmed. SNU-228 control cells showed no trend in terms of an increase (*p* = 0.5251) whereas the sunitinib-treated cells showed an increased pattern for the GR_50_ values (*p* = 0.0269, Fig. 5E). SNU-267 control cells also showed no increase (*p* = 0.6275) whereas sunitinib-treated cells showed an increasing pattern in relation to the GR_50_ (*p* = 0.0215, Fig. 6E).

Lastly, the GR_100_ value of the SNU-228 control cells showed no statistically significant increase (*p* = 0.0594), and the progression of the GR_100_ values in the cells continuously exposed to sunitinib were statistically increased (*p* = 0.0019, Fig. 5F). The GR_100_ values of the control SNU-267 cells also showed no increase (*p* = 0.5516), whereas the GR_100_ of the cells exposed to sunitinib was increased significantly (*p* < 0.0001, Fig. 6F).

Based on the current observations of changes in the resistance indicators during sunitinib treatment of SNU-228 and SNU-267 cells, the IC_50_ and GI_50_ values were observed not to reflect the resistance establishment process, while the GI_100_, GR_50_ and GR_100_ values continued to increase.

### The advantages and disadvantages of different resistance assessment indicator candidates

**Fig. 7:**
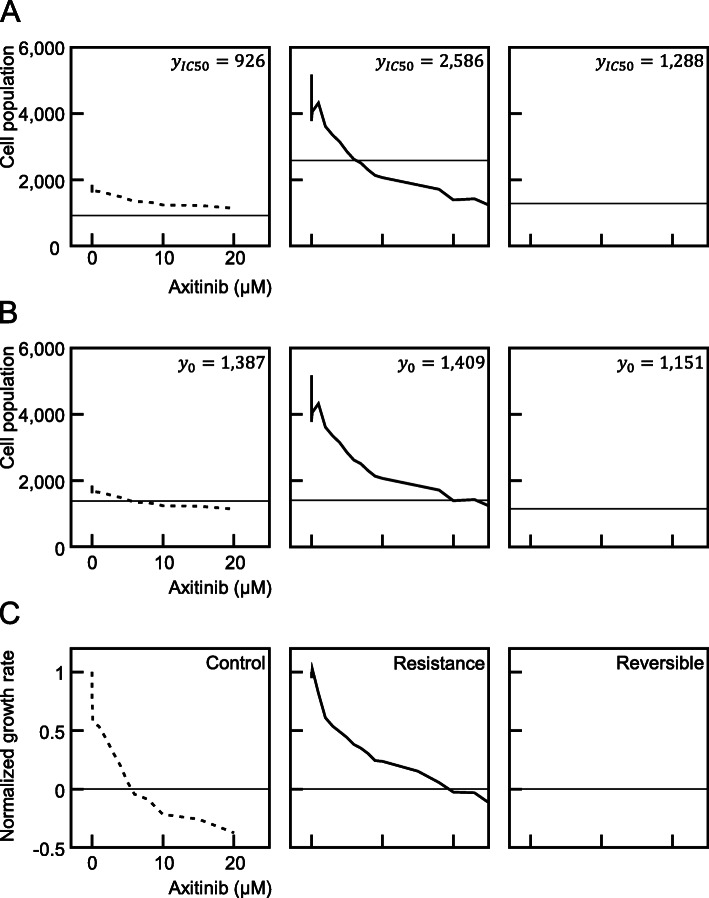
Comparison of the IC, GI, and GR parameters in evaluating anti-cancer drug resistance. The graph shown in the second column of Fig. 8A were analyzed in three respects. The same analyses were conducted for the IC_50_, GI_100_, and GR_100_. The number of cells corresponding to the IC_50_ in (**A**), GI_100_ in (**B**), and GR_100_ in (**C**) is indicated by a horizontal line. Data from the control groups not exposed to drugs are shown in the left column, the resistant groups exposed to sunitinib are shown in the middle column, and the resistance reversible groups are shown in the right column

Different resistance evaluation indicators were compared in SNU-228 cells treated with axitinib, which was one of the cross-resistance evaluations conducted in this study. When a horizontal line was drawn along the y-axis of the graph pointing to the IC_50_ (*y*_*IC*50_), it was observed that the sunitinib-resistant and reversible cells reached an IC_50_ at a certain concentration, but that the control cells did not even if the concentration of axitinib was increased. In this case, if cross-resistance was assessed using the IC_50_ levels, the sunitinib-resistant cells (IC_50_ = 6.33 μM) were found to more sensitive to axitinib than the control cells (IC_50_ > 20 μM). Reversible cells (IC_50_ = 15.67 μM) were also found to be more than twice as resistant to axitinib than sunitinib-resistant cells (Fig. 7A). This can happen because the doubling time between cell types varies.

If a horizontal line is drawn with the number of seeded cells on the y-axis (*y*_0_), it can be seen that the cross-resistance pattern is different from the IC_50_. The concentration of axitinib, which was the x-axis corresponding to the intersection of the horizontal line and the graph, indicates the GI_100_ value. Axitinib cross-resistance levels were measured at 9.48 μM in control cells, 23.19 μM (4.21-fold higher) in sunitinib-resistant cells, and 17.38 μM (3.15-fold higher) in reversible cells. The IC_50_ was found not only to be unstable in terms the value itself but also lacking in any clinical relevance, whereas the GI_100_ values was observed to be stable and to denote the clinically ineffective concentration of an anticancer drug.

When the y-axis indicates the cell population, it is difficult to make a comparison at a glance because the baselines for both the IC_50_ and GI_−_s on different graphs are different. By changing the y-axis to the cell proliferation rate (%), the GI_−_ values can be compared on the same basis. Moreover, changing the y-axis to the normalized growth rate, which takes into account the doubling time of the cell, allows for a more accurate assessment of drug resistance. In this instance, the x-axis corresponding to 0.5 of the y-axis indicates the GR_50_, and the x-axis corresponding to 0 is the GR_100_. According to Hafner et al., the GR_50_ can express the cell death effects of the drug, but if the GR_100_ is used, it will have clinical meaning, thus making it a better indicator of resistance. In evaluating the cross-resistance of axitinib with the GR_100_ in the present study, a value of 5.58 μM was calculated in control cells, 19.37 μM (3.47-fold higher) in sunitinib-resistant cells, and 17.91 μM (3.21-fold higher) in reversible cells.

### Two factors to consider when evaluating the resistance of anti-cancer drugs

**Fig. 8:**
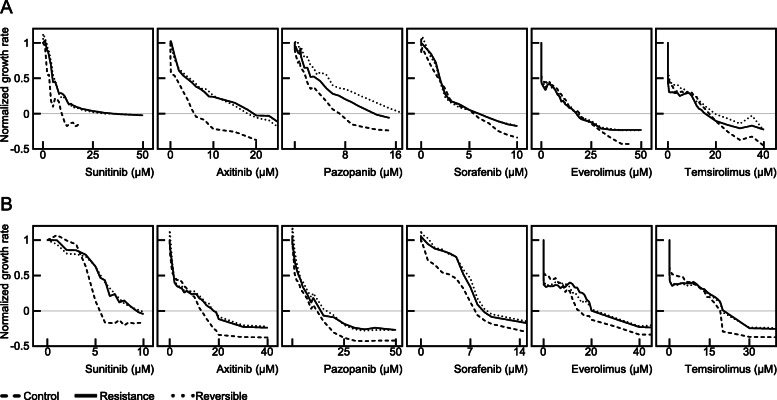
Effectiveness of six different anti-cancer drugs against sunitinib-resistant SNU-228 and SNU-267 cells. Using the SNU-228 (**A**) and SNU-267 (**B**) cell lines, three groups of cells (control, sunitinib-resistant, and resistance-reversible) were exposed to sunitinib, axitinib, pazopanib, sorafenib, everolimus and temsirolimus for 72 h and the normalized cell growth rate was then measured. This experiment was conducted three times independently

The resistance assessment indicators discussed thus far in this study cannot alone represent all of the features of resistance. The GR_100_ (or at least the GI_100_), which is claimed to be useful based on the findings of this study, was used as an indicator of drug efficiency because this is a minimum requirement for assessing anti-cancer drug effectiveness. Figure 8 shows the cross-resistance of five anti-cancer drugs in sunitinib-resistant cells. The best example of this from the present analyses was that in SNU-228 cells, the GR_100_ for everolimus was almost identical (i.e. within a 10% range) between the control cells and sunitinib-resistant cells. However, as the concentration of everolimus increased, the rate of proliferation of these two cell types changed. At the highest concentration, the cell proliferation rate forms a plateau, which is the best effect of everolimus (efficacy). At this highest concentration, the control cell proliferation decreased by 1.43 (100%) from the normalized growth rate of 1, but this reduction was only 1.23 (86%) for sunitinib-resistant cells. That is, a cross-resistance to everolimus existed in sunitinib-resistant SNU-228 cells. An explanation for this is that a complete inhibition of the proliferation of sunitinib-resistant cells indicated no cross-resistance to everolimus (0.91-fold, Table 3), but the higher concentrations of this drug produced a cross-resistance to cell death (86% compared to the control, Table 5). These patterns can vary from person to person, resulting in different conclusions in different cell types. In SNU-267 cells, the GR_100_ (efficiency) of everolimus increased by 1.35-fold and the cell death at the high concentration decreased by 92% (Tables 4 and 5). Resistance (or cross-resistance) can be assessed in this way for all of the drugs used in this study.

**Table 3:** Comparison of resistance indicator candidates for six anticancer drugs in SNU-228 cells

		Sunitinib	Axitinib	Pazopanib	Sorafenib	Everolimus	Temsirolimus
IC50	Control	10.2	> 20	7.02	> 10	19.45	19.72
	Resistant cells	11.76 (1.15)	6.33 (< 0.32)	9.22 (1.31)	2.38 (< 0.24)	11.03 (0.57)	12.21 (0.62)
	Reversible	10.04 (0.98)	15.67 (< 0.78)	9.62 (1.37)	2.95 (< 0.30)	9.3 (0.48)	10.63 (0.54)
GI50	Control	2.88	1.74	1.57	1.8	0.08	0.01
	Resistant cells	4.65 (1.61)	3.31 (1.90)	3.54 (2.25)	1.86 (1.03)	0.001 (0.01)	0.003 (0.30)
	Reversible	5.08 (1.76)	3.77 (2.17)	5 (3.18)	1.83 (1.02)	0.0006 (0.01)	0.73 (73.0)
GR50	Control	2.84	1.33	1.78	1.77	0.07	0.09
	Resistant cells	4.77 (1.68)	3.78 (2.84)	3.4 (1.91)	1.93 (1.09)	0.003 (0.04)	0.02 (0.22)
	Reversible	5.63 (1.98)	4.54 (3.41)	5.32 (2.99)	1.86 (1.05)	0.007 (0.10)	0.51 (5.67)
GI100	Control	9.48	5.51	6.89	5.41	18.96	16.72
	Resistant cells	35.1 (3.70)	23.19 (4.21)	11.94 (1.73)	6.59 (1.22)	16.59 (0.88)	14.61 (0.87)
	Reversible	25.24 (2.66)	17.38 (3.15)	16.26 (2.36)	6.17 (1.14)	17.42 (0.92)	14.05 (0.84)
GR100	Control	9.5	5.58	7.07	5.46	19.17	17.19
	Resistant cells	30 (3.16)	19.37 (3.47)	12.62 (1.79)	5.99 (1.10)	17.4 (0.91)	15.64 (0.91)
	Reversible	32.19 (3.39)	17.91 (3.21)	17.34 (2.45)	5.94 (1.09)	17.96 (0.94)	19.51 (1.13)

**Table 4:** Comparison of resistance indicator candidates for six anticancer drugs in SNU-267 cells

		Sunitinib	Axitinib	Pazopanib	Sorafenib	Everolimus	Temsirolimus
IC50	Control	5.66	7.49	4.91	6.46	8.22	11.64
	Resistant cells	5.48 (0.97)	1.5 (0.20)	3.75 (0.76)	6.34 (0.98)	0.008 (< 0.01)	0.008 (< 0.01)
	Reversible	5.61 (0.99)	1.93 (0.26)	3.51 (0.71)	6.71 (1.04)	0.33 (0.04)	0.01 (< 0.01)
GI50	Control	4.28	0.92	0.97	3.19	0.06	0.07
	Resistant cells	5.1 (1.19)	1.14 (1.24)	1.92 (1.98)	5.59 (1.75)	0.007 (0.12)	0.006 (0.09)
	Reversible	5.08 (1.19)	1.7 (1.85)	2.65 (2.73)	5.97 (1.87)	0.009 (0.15)	0.008 (0.11)
GR50	Control	4.27	1.68	1.28	4.25	0.86	2.58
	Resistant cells	5.41 (1.27)	1.62 (0.96)	3.06 (2.39)	6.11 (1.44)	0.009 (0.01)	0.01 (< 0.01)
	Reversible	5.57 (1.30)	1.72 (1.02)	3.46 (2.70)	6.7 (1.58)	0.009 (0.01)	0.0103 (< 0.01)
GI100	Control	5.45	13.15	9.45	7.67	15.02	18.92
	Resistant cells	9.12 (1.67)	17.9 (1.36)	13.85 (1.47)	8.71 (1.14)	20.2 (1.34)	20.6 (1.09)
	Reversible	9.75 (1.79)	16.85 (1.28)	17.75 (1.88)	9.43 (1.23)	22.05 (1.47)	19.9 (1.05)
GR100	Control	5.46	12.96	11.81	7.67	15.01	19.02
	Resistant cells	9.29 (1.70)	18.08 (1.40)	13.67 (1.16)	8.82 (1.15)	20.29 (1.35)	20.28 (1.07)
	Reversible	9.73 (1.78)	18.11 (1.40)	16.46 (1.39)	9.37 (1.22)	21.81 (1.45)	19.85 (1.04)

**Table 5:** Normalized growth rate reduction of SNU-228 and SNU-267 cells at the highest concentrations used of the indicated anti-cancer drugs

		Sunitinib	Axitinib	Pazopanib	Sorafenib	Everolimus	Temsirolimus
SNU-228	Control	1.13	1.37	1.24	1.34	1.43	1.45
	Resistant cells	0.93 (0.82)	1.02 (0.74)	1.06 (0.85)	1.18 (0.88)	1.23 (0.86)	1.23 (0.85)
	Reversible	0.94 (0.83)	1.06 (0.77)	0.93 (0.75)	1.17 (0.87)	1.24 (0.87)	1.24 (0.86)
SNU-267	Control	1.17	1.38	1.42	1.36	1.33	1.35
	Resistant cells	1.05 (0.90)	1.24 (0.90)	1.27 (0.89)	1.24 (0.91)	1.23 (0.92)	1.24 (0.92)
	Reversible	1.01 (0.86)	1.22 (0.88)	1.27 (0.89)	1.23 (0.90)	1.19 (0.89)	1.23 (0.91)

### Comparison with existing studies

Prior to this study, two reports had also established sunitinib-resistant cells using renal cell carcinoma cell lines and verified their cross-resistance. The major differences of those prior reports from this present study were that the cells had been continuously to sunitinib, and the resistance indicator (IC_50_) was not tracked over time but only compared at the beginning and end of the experiment. As mentioned earlier, it is recommended that information on drug resistance be viewed from a large framework, taking into account both the efficiency (GR_100_) of these agents and their relative efficacy (Table 6).

**Table 6:** Comparison between the current study findings and the results of prior studies of sunitinib resistance and cross resistance

Publication year	2013 [2]	2015 [9]	This study		This study	
Cell line	ACHN	786-O	SNU-228		SNU-267	
Age	22	58	51		43	
Sex	male	male	male		male	
Ethnicity	Caucasian	Caucasian	Korean		Korean	
Origin	metastasized tumor	primary tumor	primary tumor		primary tumor	
Cytology	adenocarcinoma	clear cell adenocarcinoma	clear cell adenocarcinoma		clear cell adenocarcinoma	
Drug exposure method	continuous	continuous	pulse		pulse	
Drug concentration	up to 10 μM	5 μM	3 μM		5.5 to 7 μM	
Resistance indicator	IC_50_	IC_50_	GR_100_	Efficacy	GR_100_	Efficacy
Fold increase						
Sunitinib	5	3.1	3.16	0.82	1.70	0.89
Axitinib			3.47	0.74	1.40	0.89
Erlotinib		4.5				
Lapatinib		1.4				
Pazopanib		1.6	1.79	0.85	1.16	0.89
Sorafenib	6	1.2	1.10	0.88	1.15	0.91
Everolimus	1	23	0.91	0.86	1.35	0.92
Temsirolimus	1		0.91	0.84	1.07	0.91

## Discussion

The tumor size (or volume) is an absolute criterion for determining cancer progression or the effectiveness of anti-cancer drugs, and tumor estimation technology is also evolving continuously [12, 13]. This is not only the case in humans, but also in the safer experimental mouse models [14]. In these in vivo animal studies, whether the tumor size is smaller after treatment is the key measure of treatment effectiveness. Notably in this regard, in vitro cell line studies conducted prior to in vivo studies in animals have traditionally utilised the IC_50_, an indicator that does not take into account the number of cells before treatment [3]. Hence, the utility of IC_50_, the GIs (50 and 100), and the GRs (50 and 100), which are all candidates for the evaluation of chemotherapy effects, was compared in this present study.

In the current analyses, trends in drug resistance assessment indicators were observed in the process of resistance development in two RCC cell lines over 900 days of sunitinib treatment. The IC_50_ and GI_50_ were found not to reflect the occurrence of resistance over time. Although the IC_50_ and GI_50_ values were sometimes higher in sunitinib-treated cells than in control cells, it is concluded that these indicators could not stably represent resistance during the whole study period. The most recently developed indicator of anti-cancer drug effectiveness, the GR_50_, was found to reflect, statistically significantly, the process of establishing drug resistance in the RCC cells. The GI_100_ and GR_100_, indicators that include clinical goals, also reliably represented the process of establishing resistance. GR_100_, an indicator that takes the doubling time of the cells into account, is preferred to GI_100_, but both of these values indicate the efficiency of anticancer drugs. In addition, when considering the maximum amount of cell death as the indicator of the overall efficacy of anti-cancer drugs, suitable systems can now be established using these parameters to assess the resistance to anti-cancer drugs.

The IC_50_, an indicator of the cytotoxicity of anticancer drugs in vitro, has now been in use for a very long time. The shortcomings of this measure have now become apparent however through the experience of researchers and continued efforts have been made to improve it. In the evaluation of drugs other than anticancer agents, there are also reports that IC_50_ values are not stable either and can be applied only in limited cases [15]. Some prior reports have indicated that there is a problem with the MTT experimental method for calculating the IC_50_, and tried to improve on this cell viability measurement method [16]. However, as highlighted several times in this current study, the IC_50_ is not now recommended for use in anti-cancer drug efficacy or resistance studies, as it lacks meaning in terms of the clinical effects of these agents. This problem also exists for the GI_50_. Even though this parameter takes into account the number of seeded cells, a reduction in the rate of proliferation by just 50% is not clinically meaningful. Hence, new parameters including the relative doubling capacity inhibition (RD) and total growth inhibition (TGI) were introduced in a prior study, which highlighted the problems with the IC_50_ and GI_50_ [17]. Another study introduced the GR_50_, which corrected for the doubling time of the cells [10].

This present study is meaningful in that it has verified recently developed anti-cancer drug effectiveness evaluation indicators and proposed pharmacologically valid and clinically effective drug efficacy evaluation methods. Recent studies have highlighted the need to design experiments in a way that better enables bench to bedside translation in a number of areas, including chemotherapeutics [18, 19]. In vitro cell research into anti-cancer and other drugs must adopt criteria that have relevance to the real world clinical environment. The efforts undertaken in this present study and others will pave the way for cell research data to be generated in a way that has a meaningful connection to potential clinical applications.

## Conclusion

It is recommended based on our current findings to use the GR_100_ to assess the efficiency of an anti-cancer drug and also the maximal cell death it causes at high concentrations as an efficacy evaluation. Importantly, both the pharmacological perspectives and clinical utility aspects of an anti-cancer drug can thereby be satisfied when assessing resistance. The effectiveness assessment of anti-cancer drugs is essentially a core component of an resistance assessment because resistance indicates a loss of drug potency. The methodologies proposed in this present study will help with future evaluations of the effectiveness, resistance, and cross-resistance of anti-cancer drugs using in vitro cell research systems.

## Supplementary Information

**Additional file 1 **: **Supplementary Table 1**. Sunitinib cell exposure conditions.

## Data Availability

Not applicable.

## Abbreviations

GI: Growth inhibition
IC: Inhibitory concentration
GR: Normalized growth rate inhibition
y_0_: Number of seeded cells on the y-axis
RD: Relative doubling capacity inhibition
RCC: Renal cell carcinoma
TGI: Total growth inhibition
