# Supplementary material for: Prospective pharmacological methodology for establishing and evaluating anti-cancer drug resistant cell lines
Source: BMC Cancer. 2021 Sep 25;21:1049. doi: 10.1186/s12885-021-08784-7 (PMC8464141; doi:10.1186/s12885-021-08784-7)
Supplement: Supplementary file 1 — Additional file 1 : Supplementary Table 1. Sunitinib cell exposure conditions. [file 12885_2021_8784_MOESM1_ESM.docx]

Supplementary Table 1. Sunitinib cell exposure conditions

| Duration of exposure | | Drug concentration (μM) | |
| --- | --- | --- | --- |
|  |  | SNU-228 | SNU-267 |
| Over 4 weeks |  | 3 | 5.5 |
| Over 2 weeks | First 48 hours | 1.5 | 2.75 |
|  | Second 48 hours | 0.75 | 1.38 |
|  | Third 48 hours | 0.38 | 0.69 |
|  | Fourth 48 hours | 0.19 | 0.34 |
|  | Fifth 48 hours | 0.09 | 0.17 |
|  | Sixth 48 hours | 0.05 | 0.09 |
|  | Seventh 48 hours | 0.02 | 0.05 |
